# Fevers

**Published:** 1904-09-03

**Authors:** 


### FEVERS.

**"Paratyphoid" Fever.**—This disease, of which a hundred or more cases have now been described, has been very recently differentiated from typhoid fever as a result of the close study which the bacteriology of the latter disease has received of late years. Its main features are described by R. Tanner Hewlett.[21] It differs mainly from true typhoid in that the organism which has been isolated, though allied to, is not identical with *B. typhosus.* In some cases, also clinically resembling mild typhoid, a true colon bacillus has been isolated from the urine. Ker[22] mentions such a series of cases reported by Burch. Widal's reaction was negative, but the serum agglutinated cultures of the colon bacillus. The "paratyphoid," or as they are sometimes called the "paracolon" bacilli are intermediate in type between the true typhoid and the colon bacillus. They have been placed in the "Gärtner" group to which also belong *B. enteritidis,* the cause of epidemic meat poisoning, *B. psittacosis,* which causes epidemic pneumonia, and the bacilli of hog-cholera and mouse-plague, *B. choleræ suum* and *B. typhi murium* respectively which are non-pathogenic to man. Hewlett furnishes a table showing the chief cultural differences between the typhoid, intermediate, and colon groups. Like *B. typhosus* the paratyphoid bacilli are multiflagellate actively motile bacilli, which refuse to stain with Gram's method, do not form spores, do not ferment lactose, and form similar growths on agar and gelatine. They ferment dextrose, producing gas as well as acid, and develop a transient not a permanent acidity in litmus milk. The colon bacillus, on the other hand, produces permanent acidity plus curdling in litmus milk, and also ferments lactose. The paratyphoid and typhoid bacilli do not form indol, but bacillus coli does. Minute cultural differences divide the paratyphoid bacilli themselves into at least two groups, the $\alpha$ and $\beta$ groups. All the symptoms and all the complications, including relapses, present in typhoid fever may be present in the paratyphoid variety. The cases are on the whole, however, less severe, and the four or five which have come to autopsy have shown an apparent absence of intestinal lesions. Widal's reaction cannot be obtained, but the blood serum is found to readily agglutinate a paratyphoid culture of the $\alpha$ or $\beta$ strain as the case may be.

**Antityphoid Inoculation.**—This subject, together with the general principles involved in protective inoculation and immunisation, is considered at some length by Professor A. E. Wright,[23] in a series of papers. In the use of preventive inoculations, careful attention must be paid to the law of ebb and flow of susceptibility. The inoculation of a vaccine is always followed by a negative phase in which the blood is impoverished in antitropic substances, and the patient is therefore more liable to contract the disease. This is succeeded by a positive phase in which the blood is flooded with newly-formed antitropic substances, and the patient consequently is very little susceptible to the disease. Finally, these transient phenomena are succeeded by a moderate positive phase with a more or less permanent diminished susceptibility. Too large an initial dose of vaccine may produce a dangerously prolonged period of increased susceptibility. Two successive smaller inoculations properly spaced, so that a relatively high permanent positive phase is attained, answer best. The amount of constitutional disturbance evoked by the inoculation does not bear any certain relation to the degree of immunity secured. The statistics bearing on the success or otherwise of antityphoid inoculations are perhaps more than most open to fallacies, but, making ample allowance for these, a careful consideration of 27 synoptical tables, most of which are furnished by the South African war, justifies the conclusion that protective inoculation diminishes the incidence of typhoid fever by at least one-half.

Scarlet Fever.—The infectivity of the peeling skin in scarlet fever, and also the value of isolation hospitals in the treatment of this disease are points which have been challenged recently. R. E. Lauder,[24] Medical Officer of Health for Southampton, disavows his belief in skin infection, and has moreover acted on this principle for more than a year in his capacity as medical superintendent of the Southampton Fever Hospital. He does not demand that isolation hospitals should be abolished, but rather that their internal administration should be modified. He believes that in scarlet fever the upper respiratory passages, especially the naso-pharynx and the tonsils, are responsible for the reception and propagation of the infection. The intricate anatomical arrangement of the upper air-passages and their accessory sinuses, together with the abundance of lymphoid, glandular, and epithelial structures present, makes this a very likely region for the reception and retention of micro-oganisms. Moreover, in scarlatina, even the mildest cases show some inflammation of the faucial mucous membrane. Previous to 1903, all cases admitted were treated in the general wards until peeling and all discharges had ceased. Since that time a much more discerning method of segregation and disinfection has been employed. Mild and convalescent cases are separated from those in an acute stage and from those suffering from discharges. Doubtful cases are isolated in an observation ward until the doubt is cleared up. Directly a case develops aural or nasal discharge, enlarged glands, etc., it is removed to a special block or pavilion. After three weeks uncomplicated cases are disinfected and removed to a special carefully disinfected pavilion, to which only similar cases are admitted. Here nose, throat, and ears are carefully douched with a disinfectant each day and the case, provided all else is well, sent out regardless of peeling at the end of the fourth week. Complications have been reduced under this method of procedure, albuminuria, adenitis, and aural and nasal discharges being much less frequent. In 1902, 208 cases were admitted and 164 discharged after an average stay in hospital of 48 days. In 1903, 353 were admitted and 325 discharged after an average stay of 34 days. Seven "return" cases occurred in each year, but the percentages of these, owing to the larger number of admissions in 1903, work out at 4·27 and 2·15 respectively. In only two of these was infection traced to patients, 204 in number, discharged peeling but without complications, while five were traced to the 88 patients discharged with complications. The above facts seem to indicate that peeling *per se* is not a sign of infectiousness. The influence of unrecognised cases probably accounts largely for the spread of diseases like scarlet fever and diphtheria in spite of compulsory notification and isolation.[25] Stress is laid by J. D. Windle[26] on the condition of the throat as an aid to diagnosis. Some deviation from the normal in the state of the throat is regarded as the one essential clinical feature common to all cases from the mildest to the most severe. The rash occurs earlier in the throat, from 24 to 48 hours before it appears on the skin. It is best seen on the faucial pillars, the uvula, soft palate, and posterior part of hard palate, though it also occurs on the tongue and the pharyngeal wall. Sore throat is the first objective symptom of scarlet fever, the onset of the illness being, according to this observer, less sudden than is commonly supposed. At first the rash consists of minute bright red, discrete, slightly conical spots, each being surrounded by a ring of erythema. Generally by the time the skin rash appears the punctate character has given place to an intense general "sealing-wax redness" of the parts. The early appearance of rash on the throat is distinctive alike in very mild and in severe cases with suppressed cutaneous rash where high fever and pronounced nervous symptoms suggest the onset of acute meningitis or pneumonia. In measles the faucial eruption which precedes the skin rash by several days, shows spots deeper in colour and much larger than scarlatinal spots and not running together to form a vivid blush. These are not to be confounded with Koplik's spots which J. C. Muir[27] finds also appear before the rash. In scarlatiniform rubella, with which confusion is very liable to arise, the spots on the fauces are larger and more papular. Discrete spots also occur on the face and forehead, but are often transitory, and the skin rash is distinctly palpable. There are also other points of distinction Mallory,[28] of Harvard University, has described and figured certain bodies found in the skin of four patients who died in the eruptive stage of scarlet fever. He suggests that they are protozoa, but nothing certain can as yet be said as to their nature or their significance. F. P. Mackie[29] gives notes of the treatment of a series of cases of scarlet fever and of diphtheria in which antistreptococcic sera of different strains were employed. All the cases were extremely severe. Antitoxin was also given to the diphtheria cases. Since, in the writer's view, cases of diphtheria in which streptococci also are found form the most severe types, treatment with the combined sera seems reasonable, but its value is not yet established.. In scarlet fever antistreptococcic serum, if given *early*, will possibly be of distinct value, though it is difficult to say beforehand which cases will be benefited. Grunbaum[30] has produced a scarlatinal tonsillitis in a chimpanzee by rubbing the throat with a swab obtained from the throat of a patient suffering from scarlet fever.

[21] Practitioner, January. [22] Ibid., March, p. 343. [23] Ibid., Jan., Feb., and March, [24] Lancet, March 12 and March 26, p. 882. [25] Ibid., June 11, p. 1666. [26] Clin. Jour., May 18. [27] Lancet, June 11. [28] Med. Rec., Feb. 27, p. 337. [29] Lancet, Feb. 20. [30] Brit. Med. Jour., April 9.

(*To be continued.*)

